# Regional Fibronectin and Collagen Fibril Co-Assembly Directs Cell Proliferation and Microtissue Morphology

**DOI:** 10.1371/journal.pone.0077316

**Published:** 2013-10-08

**Authors:** Carlos A. Sevilla, Diane Dalecki, Denise C. Hocking

**Affiliations:** 1 Department of Biomedical Engineering, University of Rochester, Rochester, New York, United States of America; 2 Department of Pharmacology and Physiology, University of Rochester, Rochester, New York, United States of America; University of California, Berkeley, United States of America

## Abstract

The extracellular matrix protein, fibronectin stimulates cells to self-assemble into three-dimensional multicellular structures by a mechanism that requires the cell-dependent conversion of soluble fibronectin molecules into insoluble fibrils. Fibronectin also binds to collagen type I and mediates the co-assembly of collagen fibrils into the extracellular matrix. Here, the role of collagen-fibronectin binding in fibronectin-induced cellular self-assembly was investigated using fibronectin-null fibroblasts in an in vitro model of tissue formation. High resolution, two-photon immunofluorescence microscopy was combined with second harmonic generation imaging to examine spatial and temporal relationships among fibronectin and collagen fibrils, actin organization, cell proliferation, and microtissue morphology. Time course studies coupled with simultaneous 4-channel multiphoton imaging identified regional differences in fibronectin fibril conformation, collagen fibril remodeling, actin organization, and cell proliferation during three-dimensional cellular self-assembly. Regional differences in cell proliferation and fibronectin structure were dependent on both soluble fibronectin concentration and fibronectin-collagen interactions. Fibronectin-collagen binding was not necessary for either fibronectin matrix formation or intercellular cohesion. However, inhibiting fibronectin binding to collagen reduced collagen fibril remodeling, decreased fibronectin fibril extension, blocked fibronectin-induced cell proliferation, and altered microtissue morphology. Furthermore, continual fibronectin-collagen binding was necessary to maintain both cell proliferation and microtissue morphology. Collectively, these data suggest that the complex changes in extracellular matrix and cytoskeletal remodeling that mediate tissue assembly are driven, in part, by regional variations in cell-mediated fibronectin-collagen co-assembly.

## Introduction

Tissue self-assembly describes the directed organization of cells into multicellular structures [1,2]. Morphogenetic studies of embryonic development have identified self-assembled multicellular structures, including tubes, invaginations, buds, and branches, that function as basic building blocks for tissues and organs [3,4,5,6,7]. In turn, repeating combinations of various self-assembled structures give rise to the form and complexity observed in distinct tissues and organs [6]. The formation of self-assembled tissues requires cell-extracellular matrix (ECM) interactions to stimulate cell cohesion, induce cell function, and maintain structural geometry [8]. During tissue morphogenesis, ECM proteins can function solely as passive structural supports that serve to organize or segregate cells within tissues, or they can play active signaling roles to direct cell behaviors, such as cell proliferation [9]. To generate complex three-dimensional (3D) tissue geometries, the ECM must shift between passive and active states in a tightly regulated process [10]. Factors that coordinate these transitions in order to organize cells into functional tissues remain largely unknown.

Fibronectins are large ECM glycoproteins that play an important role in early developmental processes, including gastrulation [11], branching morphogenesis [12], and cardiac patterning [13]. In the body, cells continuously assemble soluble fibronectin molecules into insoluble ECM fibrils [14,15]. In turn, the fibrillar ECM form of fibronectin can stimulate cell behaviors critical to tissue formation and function, including cell proliferation [16], migration [17], contraction [18], and cell cohesion [19,20,21]. Fibronectin also binds to several other ECM proteins, including collagen type I [22] and plays a central role in ECM organization. Fibronectin matrix assembly mediates collagen fibril deposition into the ECM [23,24] and increases the tensile strength of collagen-based tissue constructs [25]. The interaction of fibronectin with collagen is necessary for ECM fibronectin-stimulated cell migration and enhanced cell contractility [26], suggesting that co-assembly of fibronectin and collagen fibrils influences ECM fibronectin function.

To investigate whether fibronectin-collagen interactions play a role in fibronectin-stimulated cellular self-assembly, we utilized a recently developed model of self-assembled, 3D microtissue, which combines compliant polymerized collagen type I substrates with the cell-mediated assembly of fibronectin matrix fibrils [21]. High resolution, two-photon microscopy was combined with second harmonic generation imaging to examine spatial and temporal relationships among fibronectin and collagen fibrils, actin organization, cell proliferation, and microtissue morphology. Fibronectin-null mouse embryonic fibroblasts (FN-null MEFs), cultured in serum- and fibronectin-free media, were utilized in this study. FN-null MEFs polymerize exogenously-added fibronectin into dense, ECM fibrils via a process indistinguishable from that of fibronectin-expressing cells [16]. Thus, this model permits tight control over the amount of fibronectin to which the cells are exposed, and the initiation and timing of the matrix assembly process. We provide evidence that the cell-mediated co-assembly of collagen and fibronectin fibrils shifts ECM fibronectin function from a structural role that mediates cell cohesion to an active, proliferative role. Further, we demonstrate temporal control of cell proliferation and 3D microtissue shape using a fibronectin-binding peptide that inhibits the binding of fibronectin to collagens I [26]. Thus, spatial and temporal variations in ECM fibronectin-collagen co-assembly provide a means to shape the growth and morphology of tissues during tissue formation and regeneration.

## Materials and Methods

### Proteins, Reagents, and Cells

Fibronectin was purified from outdated human plasma (American Red Cross, Rochester, NY) using gelatin-Sepharose (GE Healthcare Life Sciences, Piscataway, NJ) affinity chromatography [27]. Collagen type I was extracted from rat-tail tendons using acetic acid and then precipitated with NaCl, as described previously [21]. The His-tagged peptides, R1R2 and III-11C were expressed in *Escherichia coli*, purified over Ni-Sepharose, and dialyzed extensively against PBS, as described previously [28]. The 76-mer R1R2 peptide encompasses amino acids Gly^195^-Thr^253^ from the bacterial adhesin protein, SFS [28,29]. R1R2 binds to fibronectin [29] and inhibits the binding of fibronectin to native and denatured collagens I, II, and III [26]. The concentration of purified peptides was determined using a BCA assay (Pierce). Peptides were filter-sterilized and purity was assessed by SDS-polyacrylamide gel electrophoresis. Peptides were stored in aliquots at -80°C. Antibodies and their sources are as follows: Alexa Fluor^594^-conjugated anti-BrdU mAb, Alexa Fluor^488^-labeled phalloidin, Alexa Fluor^647^-conjugated goat anti-rabbit IgG, (Invitrogen, Carlsbad, CA); polyclonal anti-fibronectin (Sigma). Tissue culture supplies were from Corning/Costar (Cambridge, MA). Unless otherwise indicated, chemical reagents were from Sigma.

Mouse embryonic fibronectin-null fibroblasts (FN-null MEFs) (provided by Dr. Jane Sottile, University of Rochester, Rochester, NY) [16] were cultured on collagen type I-coated dishes at 37°C and 8% CO_2_ using a 1:1 mixture of Aim V (Invitrogen) and Cellgro® (Mediatech, Herndon, VA). These media do not contain fibronectin and do not require serum supplementation [16]. Thus, no exogenous source of fibronectin is present during routine culture. FN-null MEFs express α1, αv, α5, β1, and β3 integrin subunits, and do not express α2 and α4 integrin subunits [16]. Both collagens I and III are synthesized by FN-null MEFs [26].

### Microscopy

Native collagen gels were prepared as described [21] by mixing 4X concentrated Dulbecco’s modified Eagle’s medium (DMEM; Life Technologies), collagen, and 1X DMEM on ice such that the final mixture contained 1 mg/ml collagen and 1X DMEM. FN-null MEFs were seeded on polymerized collagen gels (5.26 x 10^4^ cells/cm^2^) in 35 mm tissue culture dishes. Four hours after seeding, fibronectin (25 or 100 nM) was added to wells and cells were incubated for 2, 4, or 6 days. In some experiments, fibronectin was pre-incubated for 60 min with the peptides R1R2 or III-11C (62.5 nM or 2.5 µM), or an equal volume of the vehicle control, PBS.

To visualize actively proliferating cells, bromodeoxyuridine (BrdU; 100 µM; BD Biosciences) was added to wells 4 h prior to fixation. Cells were fixed with 1:1 acetone/methanol (-20°C) or 4% paraformaldehyde (37°C) and permeabilized with 0.05% Triton X-100. Fibronectin was immunolabeled overnight using anti-fibronectin polyclonal antibodies. Cell nuclei, actin, BrdU, and fibronectin were visualized using 4’,6-diamidino-2-phenylindole (DAPI; 60 nM), Alexa^488^-labeled phalloidin (1:200), Alexa^594^-labeled anti-BrdU monoclonal antibody (1:200), and Alexa^647^-labeled goat anti rabbit antibody (1:200), respectively.

Immunofluorescence images were collected using an Olympus Fluoview 1000 AOM-MPM multiphoton microscope equipped with a 25X, 1.05 NA water immersion lens (Olympus). Fluorophores were simultaneously excited at 780 nm using a femtosecond Mai Tai HP Deep See Ti:Sa laser (Spectra-Physics, Mountain View, CA, USA). DAPI, Alexa^488^, and Alexa^594^ emitted fluorescence were separated using a 505 nm long-pass dichroic mirror and filtered using a 460 nm-bandpass filter (#FF01-460-80, Semrock), a 519 nm-bandpass filter (#BA495-546, Olympus), and a 609 nm-bandpass filter (#FF01-609/54-25, Semrock), respectively. Alexa^647^ emitted fluorescence was separated using a 750 nm short-pass dichroic mirror and filtered with a 670 nm-bandpass filter (#FF01-670/30-25, Semrock).

Cross-reactivity was assessed for each secondary antibody utilized. Briefly, Alexa-labeled goat anti-rabbit, goat anti-mouse, and donkey anti-goat secondary antibodies were used to stain non-target primary antibodies, and fluorescence was imaged using two-photon microscopy. Antibody cross-reactivity did not occur with any of the Alexa Fluor-conjugated secondary antibodies tested. Single-labeled controls were also used to assess channel crosstalk [30]. FN-null MEFs, adherent to polymerized collagen gels, were incubated with fibronectin (25 nM) for 4 days. Fibronectin was detected using an anti-fibronectin polyclonal antibody followed by Alexa Fluor^488^-, Alexa Fluor^594^-, or Alexa Fluor^647^-conjugated goat anti-rabbit IgGs, and images were obtained. Crosstalk occurred from the Alexa Fluor^488^ channel into the Alexa Fluor^594^ channel. To correct for 488- to 594-channel crosstalk in 4-channel imaging, Alexa Fluor^488^ images (actin) were subtracted from their respective Alexa Fluor^594^ images (BrdU) using the image calculator function of ImageJ (NIH, Bethesda, Maryland) [31,32].

Collagen was visualized with second-harmonic generation microscopy [33] using the Olympus Fluoview 1000 AOM-MPM multiphoton microscope described above. Collagen second-harmonic fluorescence was separated using a 505 nm long-pass dichroic mirror and filtered using a 390 nm-bandpass filter (#FF01-390/40-25, Semrock). Image brightness and contrast were enhanced offline using Adobe Photoshop®. Images within each figure were brightness and contrast enhanced to the same extent.

Aberrations in the 750 nm short-pass dichroic mirror resulted in Alexa Fluor^647^ images that were unaligned in the x-y direction as compared to images collected using the 505 nm long-pass dichroic mirror (DAPI, Alexa Fluor^488^, and Alexa Fluor^594^). To correct the aberration, Alexa Fluor^488^ emitted fluorescence was collected with both the 505 nm long-pass dichroic mirror (target image) and the 750 nm short-pass dichroic mirror (reference image). Image alignment was performed offline using ImageJ. Target and reference images were merged and the aberration quantified by measuring the lateral and vertical distance between identical landmarks in the merged image. Alexa Fluor^647^ images were then aligned using the translate function in ImageJ with the calculated lateral and vertical aberrations as the respective x- and y-offsets [34].

### Image Analysis

The spatial organization of proliferating cells was determined from 4-channel images (DAPI, BrdU, actin, fibronectin) using ImageJ. To determine the relative distance of a BrdU-positive cell from the microtissue centroid (r_a_/r_b_), actin and BrdU image stacks were merged. r_a_/r_b_ was calculated as the ratio between the straight-line distance from the microtissue centroid to the BrdU-positive cell (r_a_) and the straight-line distance from the microtissue centroid to the perimeter of the microtissue (r_b_). To determine the microtissue centroid, actin image slices corresponding to the microtissue-collagen substrate interface were thresholded using ImageJ’s Modified IsoData method. The centroid of the microtissue was then calculated from the thresholded actin image using ImageJ’s image moments calculator. The perimeter of a microtissue was defined by actin staining. The ratio, r_a_/r_b_ was calculated for all proliferating cells throughout the vertical height of a microtissue. Nine total multicellular structures per condition (day 2 and day 4 with 25 or 100 nM fibronectin) from 3 independent experiments were used to calculate r_a_/r_b_.

A theoretical calculation of the expected distribution of r_a_/r_b_ for randomly proliferating cells within a circle was also performed. The circle diameter was defined as 1 unit, and the center of the circle was set at the origin of a Cartesian coordinate system (x =0, y =0). The x-coordinate and y-coordinate for 200 ‘proliferating’ cells within the circle were randomly assigned using a random number generator. The distance from the origin of the circle to each ‘proliferating’ cell (r_a_) was calculated using Pythagorean’s theorem. The distance from the origin of the circle to the edge of the circle (r_b_) was equal to the radius of the circle (0.5 units). The percent of ‘proliferating’ cells present within a 0.1 unit bin range were recorded on a histogram. The analysis was repeated ten times and the mean percent ‘proliferating’ cells within r_a_/r_b_ ≤ 0.5 and r_a_/r_b_ > 0.5 ± the standard error of the mean (SEM) was calculated using GraphPad Prism software (La Jolla, CA).

To determine the total number of proliferating cells in each microtissue, BrdU image stacks were loaded into ImageJ and BrdU-positive nuclei were counted manually using ImageJ’s cell counter function. Total cell number in microtissues was determined similarly using DAPI image stacks. The percent BrdU-positive cells for each microtissue was calculated as the number of BrdU-positive cells divided by total cell number.

### Cell Proliferation

Monodispersed FN-null MEFs were seeded on polymerized collagen gels (5.26 x 10^4^ cells/cm^2^) in 48-well tissue culture plates. Cells were allowed to adhere to the collagen substrate for 4 h and then treated with fibronectin (25 or 100 nM; 12.5 or 50 μg/ml) in the absence or presence of either R1R2 or a control peptide III-11C (62.5 nM or 2.5 µM for 25 and 100 nM fibronectin, respectively) [26]. For some experiments, fibronectin-treated cells were incubated for 3 days to establish microtissues. Media were then removed and replaced with fresh media containing R1R2- or III-11C-treated fibronectin. For all experiments, fibronectin and peptides were preincubated for 60 min. Cells were incubated for up to 6 days at 37°C and 8% CO_2_. Bright-field images were obtained using an Olympus BX60 microscope and photographed using a QImaging MicroPublisher 3.3 RTV digital camera. Cell number was determined using 3-(4,5-dimetylthiazol-2-yl)-2,5-diphenyltetrazolium bromide (MTT) (USB, Cleveland, OH), as previously described [21]. Growth assay measurements are expressed as mean ± SEM and represent 1 of at least 3 separate experiments performed in triplicate.

*Statistical Analysis* - Statistical comparisons were performed using either one-way analysis of variance (ANOVA) followed by Tukey’s post-test or Student’s t-test using GraphPad Prism software. Results were considered statistically significant when *p*<0.05.

## Results

### Dynamics of Fibronectin, Actin, and Proliferating Cells within Microtissues

**Figure 1:**
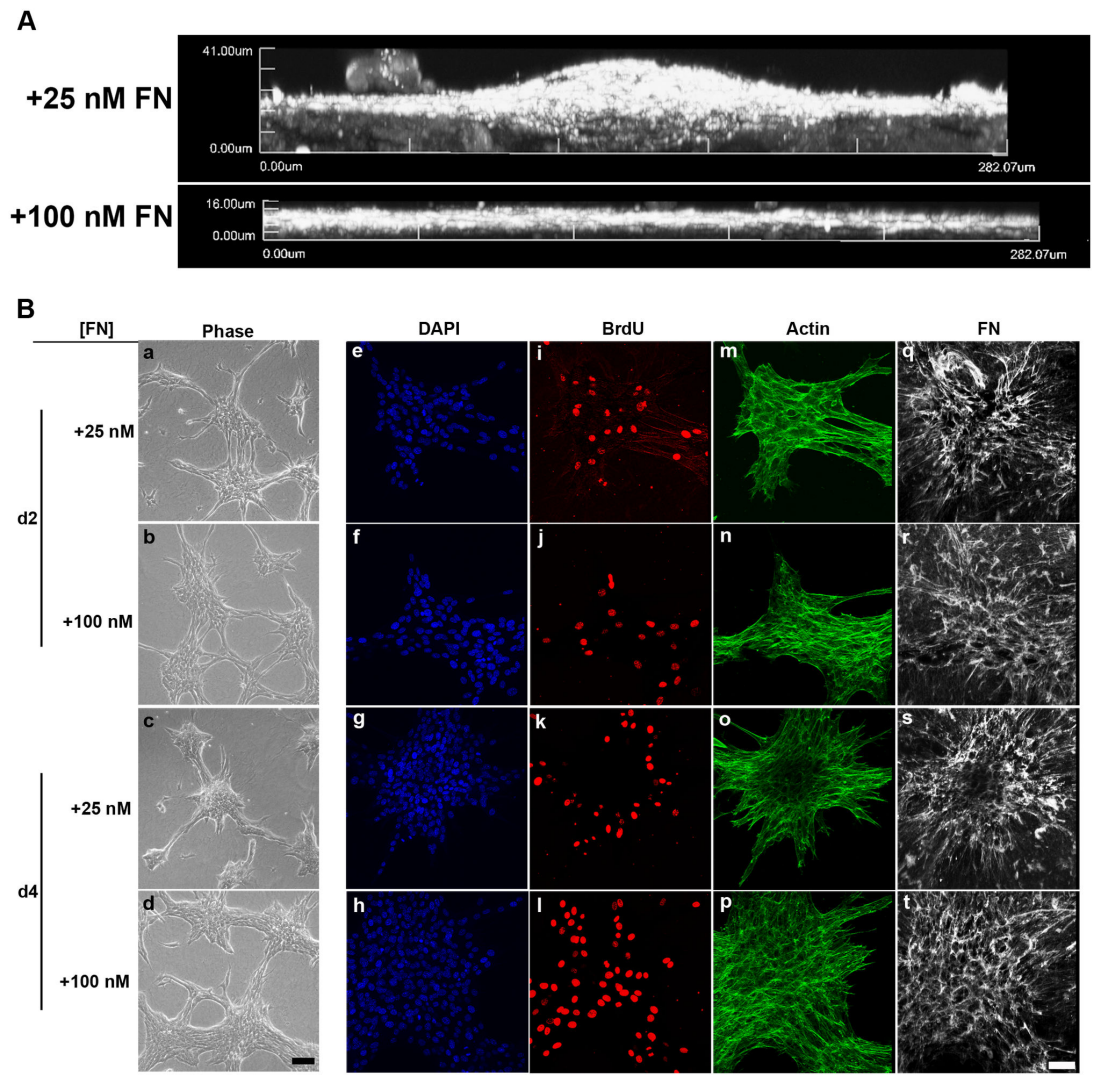
Spatial and temporal changes in cell proliferation, actin organization, and fibronectin matrix. FN-null MEFs adherent to native collagen type I gels were treated with 25 or 100 nM fibronectin (FN). (A) After a 6-day incubation, samples were immunostained for fibronectin. Z-slices (1-μm step size) were reconstructed in 3D and projected along the x-y plane. Images represent 1 of 3 experiments performed in triplicate. (B) After either a 2- or 4-day incubation, cells were processed for immunofluorescence microscopy. DAPI, BrdU, actin, and FN images were collected simultaneously along the z-axis at 1-μm intervals using two-photon microscopy. Z-slices corresponding to the microtissue-collagen interface were projected along the z-plane using ImageJ. Scale bar, 50 µm. Images represent 1 of 3 experiments performed in triplicate.

The active assembly of fibronectin fibrils by cells adherent to native collagen substrates stimulates cell proliferation and induces cells to self-assemble into multicellular structures [21,35]. These 3D structures reach a vertical height of ~50 μm five days after a single fibronectin treatment [21]. Microtissue height exhibits a biphasic response to fibronectin concentration, with peak height occurring in response to 25 nM fibronectin [21]. In the present study, 4-channel multi-photon microscopy was used to simultaneously visualize and compare spatial patterns of fibronectin fibrils, actin filaments, and proliferating cells during microtissue assembly. FN-null MEFs, cultured in serum-free media were utilized in this study in order to precisely control the timing and amount of fibronectin added to cells. Additionally, high (100 nM) and low (25 nM) concentrations of fibronectin were applied in order to produce two distinct microtissue morphologies [21]. To clearly illustrate these different morphologies, fibronectin-stained, 3D reconstructed images of day 6 microtissues were projected perpendicular to the imaging plane, and are shown in Figure 1A. FN-null MEFs treated with 25 nM fibronectin produced adherent 3D multicellular aggregates characterized by central dome-shaped cores that extended onto the collagen substrate (Figure 1A). In contrast, cells treated with 100 nM fibronectin formed dense, sheet-like structures with broad surface areas (Figure 1A), having an average vertical height of ~20 μm [21]. In the absence of fibronectin, cells remain dispersed and rounded on the native collagen substrate and do not proliferate [21].

Time course studies showed that 2 days after addition of 25 nM or 100 nM fibronectin, cells had assembled into discontinuous cell sheets (Fig. 1Ba, b). Proliferating cells were observed throughout the cell sheets (Fig. 1Be, f, i, j). At this time point, actin stress fibers were prominent (Fig. 1Bm, n) and an abundant fibrillar fibronectin matrix was present that extended across the surface of the collagen substrate (Fig. 1Bq, r). By day 4, cell sheets induced by 25 nM fibronectin had reorganized to produce the dome-shaped aggregates (Fig 1Bc, g). The central core of these 3D aggregates contained densely packed, non-proliferating cells (Fig. 1Bg, k), cortical actin (Fig. 1Bo), and pericellular fibronectin (Fig. 1Bs). In contrast to the central core, peripheral regions of the 3D aggregates contained proliferating cells (Fig. 1Bk), actin stress fibers (Fig. 1Bo), and fibrillar fibronectin that radiated out from the central core (Fig 1Bs). In response to 100 nM fibronectin, cell sheets increased in cell density between day 2 and day 4 (Fig. 1Bb, d), but did not transition into the dome-shaped 3D structures. Actin stress fibers and fibrillar fibronectin fibrils were observed throughout the cell sheets (Fig. 1Bp, t). On day 6, structures formed in response to 25 nM or 100 nM fibronectin appeared identical to their respective day 4 structures (not shown).

**Figure 2:**
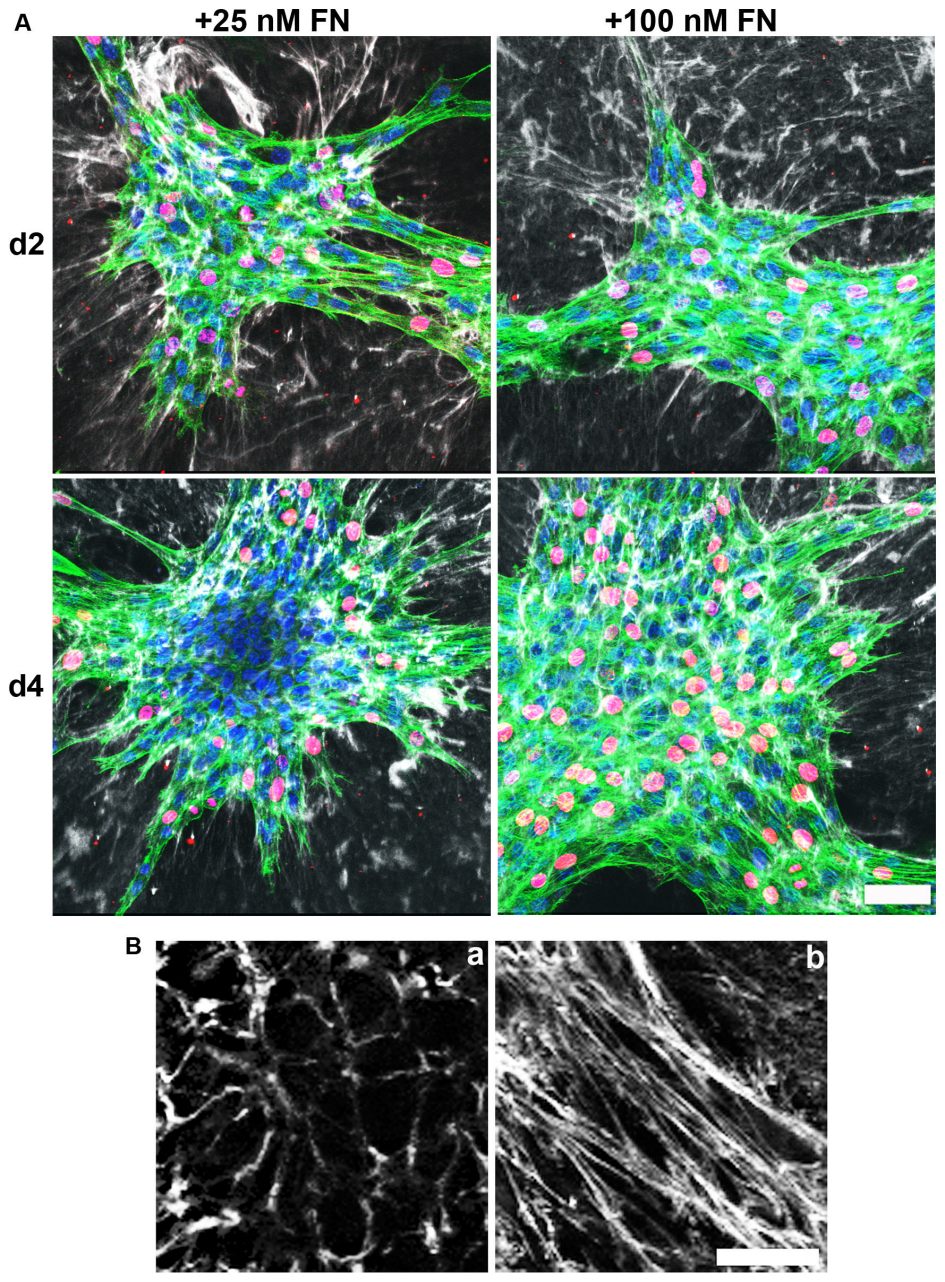
Merged 4-channel images of Day 2 and Day 4 microtissues. Collagen-adherent FN-null MEFs were treated with 25 or 100 nM fibronectin (FN). After a 2- or 4-day incubation, cells were processed for immunofluorescence microscopy. (A) DAPI (blue), BrdU (red), actin (green), and FN (white) images were collected simultaneously as described in the legend to Figure 1. Z-slices corresponding to the microtissue-collagen interface were projected along the z-plane, merged, and aligned using ImageJ. Scale bar, 50 µm. Images represent 1 of 3 experiments performed in triplicate. (B) Representative FN staining obtained from the core (a) and periphery (b) of day 4 microtissues formed in response to 25 nM FN. Note the different organizational patterns of pericellular (a) and extended (b) fibronectin fibrils. Scale bar, 100 μm.

Merged, 4-channel images are shown in Figure 2. The discontinuous cell sheets formed on day 2 in response to either fibronectin concentration showed similar patterns of co-localized actin stress fibers and fibronectin fibrils, with actively proliferating cells distributed throughout the multicellular structures (Figure 2A; d2, 25 and 100 nM FN). Similar patterns of actin stress fibers, fibronectin fibrils, and proliferating cells were observed on day 4 in response to 100 nM fibronectin (Figure 2A; d4, 100 nM FN). In contrast, 3D aggregates formed on day 4 in response to 25 nM fibronectin showed regional differences in actin and fibronectin staining with cortical actin and pericellular fibronectin co-localized within the compact, central core (Figure 2A; d4, 25 nM FN). Extended, radially aligned fibronectin fibrils and actin stress fibers projected from the edge of the core towards the periphery, where proliferating cells were observed (Figure 2A; d4, 25 nM FN). Higher magnification images obtained from the central and peripheral regions of the 3D aggregate (d4, 25 nM FN) demonstrate distinct organizational patterns of pericellular (Fig. 2Ba) and extended (Fig. 2Bb) fibronectin fibrils, respectively.

To quantitatively assess whether regional differences in cell proliferation occurred as a function of soluble fibronectin concentration, the distance of each proliferating, BrdU-positive cell from the geometric center of the microtissue was measured and the ratio, r_a_/r_b_, was calculated. A ratio value of 0 or 1.0 corresponds to a proliferating cell located on the centroid or edge of a multicellular structure, respectively. A theoretical calculation of the expected distributions of r_a_/r_b_ values for randomly ‘proliferating’ cells within a circle was performed in parallel. The theoretical mean percent BrdU-positive cells within 0 ≤ r_a_/r_b_ ≤0.5 and r_a_/r_b_ > 0.5 for randomly proliferating cells within a circle was 26% ± 0.7% and 74% ± 1%, respectively.

**Figure 3:**
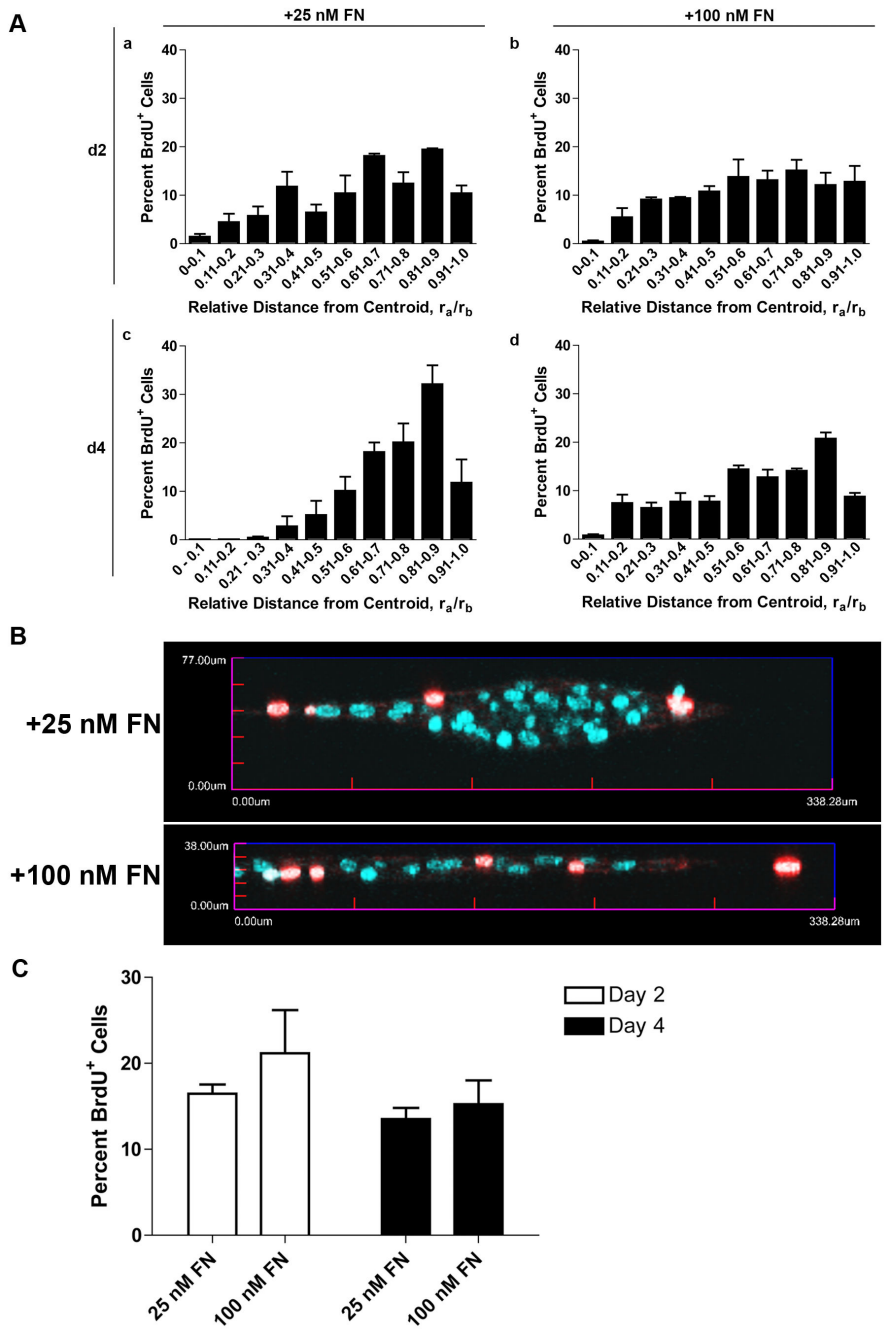
Spatial and temporal analysis of fibronectin-induced cell proliferation. Collagen-adherent FN-null MEFs were treated with 25 nM or 100 nM fibronectin (FN) for either 2 or 4 days, and then processed for immunofluorescence microscopy. Four channel images of fibronectin, actin, BrdU, and DAPI staining were obtained and processed to quantify the spatial distribution of proliferating cells in microtissues. (A) The relative distance of all BrdU-positive cells from the centroid (r _a_/r_b_) was calculated. Data are grouped to display the frequency of occurrence of proliferating cells from the centroid (r _a_/r_b_ = 0) to the periphery (r_a_/r_b_ = 1). Data are presented as the mean percent of BrdU-positive cells ± SEM of 3 independent experiments. (B) Representative x-y projections showing BrdU (red) and DAPI (blue) staining on day 4. (C) Total numbers of proliferating and non-proliferating cells were determined from BrdU and DAPI staining. Data are presented as mean percent BrdU-positive cells + SEM of 3 experiments performed in triplicate.

Proliferating cells were present in all r_a_/r_b_ bins for day 2 (Fig. 3Aa, b) and day 4 cell sheets (Fig. 3Ad). The percentage of BrdU-positive cells located within 0 ≤ r_a_/r_b_ ≤0.5 and r_a_/r_b_ > 0.5 for day 4 cell sheets formed in response to 100 nM fibronectin was 30% ± 1% and 69% ± 1%, respectively, confirming the random appearance of proliferating cells in the immunofluorescence images (Figure 2A). In contrast, proliferating cells were shifted to the outer r_a_/r_b_ bins of 3D aggregates formed in response to 25 nM fibronectin (Fig. 3Ac); the mean percentages of BrdU-positive cells within 0 ≤ r_a_/r_b_ ≤0.5 and r_a_/r_b_ > 0.5 of 3D aggregates on day 4 were 8% ± 3% and 92% ± 3%, respectively. As a final verification of this spatial organization of proliferating cells, images collected on day 4 were reconstructed in 3D and the center z-slices were projected along the x-y plane. Proliferating cells preferentially localized to the peripheral regions of 3D aggregates (Figure 3B; +25 nM FN). In contrast, proliferating cells were randomly distributed throughout the cell sheets (Figure 3B; +100 nM FN). The reduction in cell proliferation found within the central core of the 3D aggregates was not due to an overall decrease in the rate of cell proliferation, as the percent of BrdU-positive cells did not decrease between days 2 and 4 (Figure 3C; 25 nM FN). Moreover, the percentage of proliferating cells was not significantly different between fibronectin concentrations (Figure 3C). Taken together, these data demonstrate the development of regional variations in the location of actively proliferating cells that is dependent on soluble fibronectin concentration.

### Fibronectin-mediated Collagen Organization, Cell Proliferation, and Cellular Self-assembly

**Figure 4:**
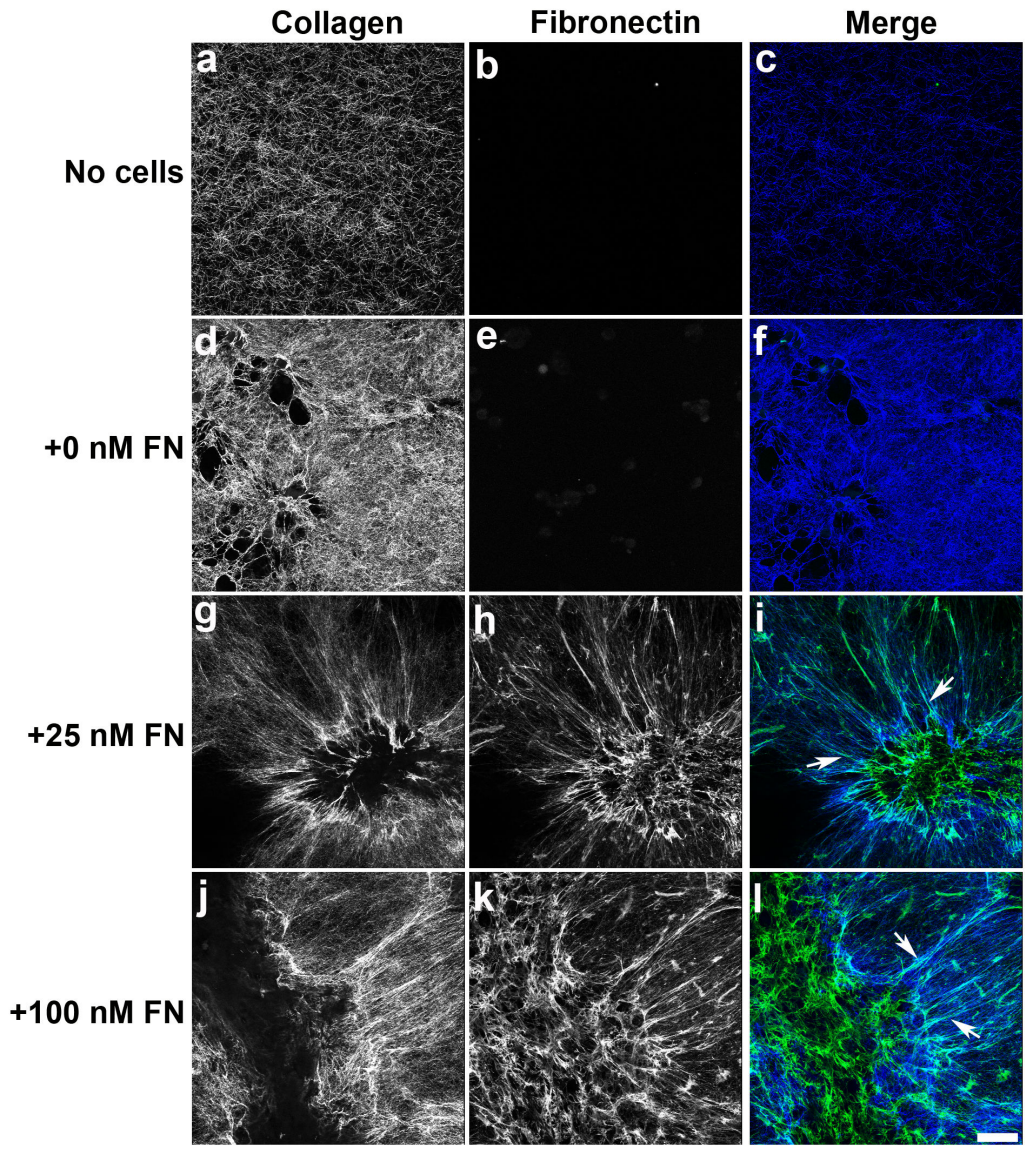
Collagen and fibronectin fibril co-localization within cell networks. Collagen-adherent FN-null cells were incubated for 2 days in the absence (0 nM FN; d-f) or presence of either 25 nM (g-i) or 100 nM (j-l) fibronectin (FN). Also included are images of polymerized collagen gels incubated for 2 days in the absence of both cells and FN (a-c). Collagen (a,d,g,j) was visualized using second harmonic generation microscopy. FN (b,e,h,k) was visualized using an anti-FN pAb followed by Alexa^488^-conjugated anti-rabbit IgG. FN and collagen were simultaneously visualized along the z-axis at 1-μm intervals using two-photon microscopy and then projected onto the z-plane using ImageJ. Note that images were collected at and above the collagen surface, and do not include images collected below the surface. Merged images of collagen (blue) and FN (green) staining are shown (c,f,i,l). Co-localized signals produce cyan (arrows). Scale bar, 50 μm.

Two photon microscopy and second harmonic generation imaging were utilized to visualize fibronectin and collagen fibril organization within the discontinuous cell sheets that formed 2 days after fibronectin addition, prior to the observed spatial segregation of proliferating cells. In the absence of fibronectin, minimal cell-mediated collagen fibril reorganization was observed (Figure 4a, d). In contrast, addition of fibronectin to collagen-adherent cells triggered the reorganization of collagen fibrils into long, thick bundles (Figure 4g, j). Merged, z-stack images of collagen and fibronectin (Figure 4h, k) staining collected immediately at and above the polymerized collagen gel interface showed extensive co-localization of extended collagen and fibronectin fibrils radiating away from the organized cell networks (Figure 4i, l; arrows). Collagen fibrils were largely absent from the very center of cell sheets, while fibronectin fibril staining was prominent (Figure 4i, l).

**Figure 5:**
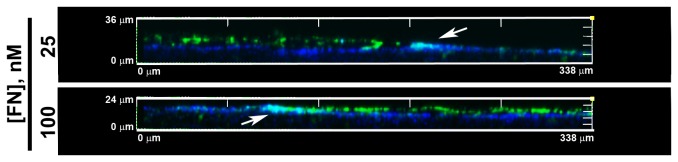
Collagen-fibronectin co-localization at the microtissue-substrate interface. Representative x-y projections of 2-day microtissues formed in response to either 25 nM or 100 nM fibronectin (FN). FN (green) and collagen (blue) were visualized as described in the legend to Figure 4. Z-slices (1-μm step size) were collected beginning below the collagen surface and extending above the microtissue. Images were reconstructed in 3D and then projected along the x-y plane. Arrows denote areas of co-localization (cyan). Note the non-overlapping FN and collagen signals on the apical and basal surfaces, respectively.

Side projection images of the day 2 cell sheets confirm the presence of distinct regions where collagen and fibronectin fibrils colocalized (Figure 5). Z-slice images obtained at 1-μm intervals beginning this time below the collagen gel interface and ending above the cell network surface were reconstructed in 3D and projected along the z-plane. Dense nodes of collagen and fibronectin co-localization were prominent at the edges of the individual cell sheets (Figure 5, arrows; cyan). At both fibronectin concentrations, collagen-free fibronectin fibrils were observed on the apical surfaces of the cell sheets (Figure 5; green), while the collagen substrate was clearly visible below the fibronectin layer (Figure 5; blue). These studies demonstrate regional variations in ECM fibronectin and collagen co-assembly within self-organizing cell sheets. These regional differences in ECM organization occur prior to the spatial shift in cell proliferation (Figure 3).

**Figure 6:**
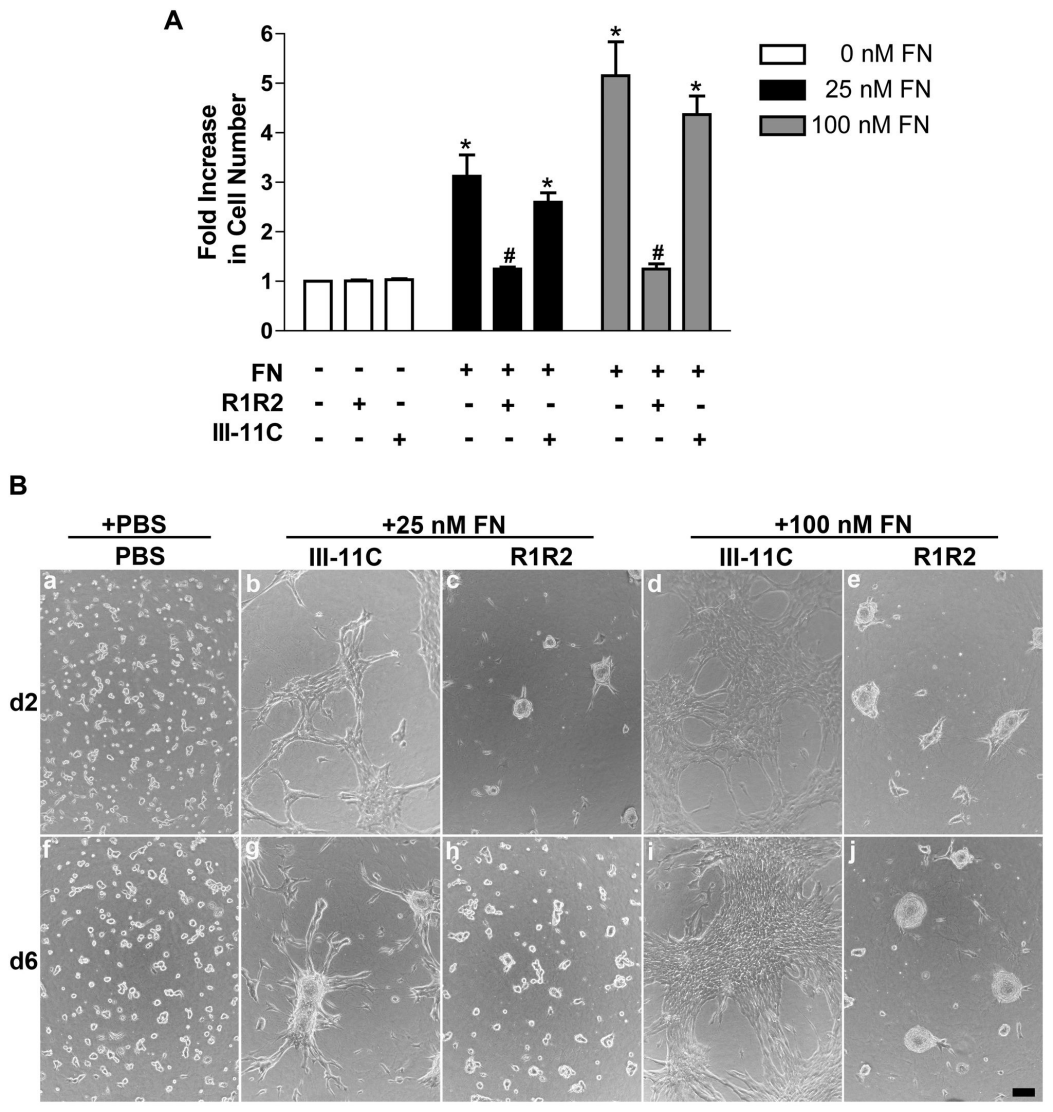
Cell proliferation and microtissue morphology require fibronectin-collagen binding. Collagen-adherent FN-null MEFs were treated with PBS (0 FN) or fibronectin (25 or 100 nM) in the absence or presence of R1R2, the control peptide III-11C, or an equal volume of the vehicle, PBS. (A) Following a 6-day incubation, cell number was determined using MTT. Data are presented as fold increase in cell number versus non-treated (+PBS/PBS) controls + SEM of 3 experiments performed in triplicate. *Significant vs. +PBS/PBS, *p*<0.001 (ANOVA). ^#^Significant vs. respective +FN/PBS and +/III-11C, *p*<0.001 (ANOVA). (B) Representative phase contrast images collected on days 2 and 6 of culture. Images represent 1 of 3 experiments performed in triplicate. Scale bar, 50 μm.

To determine whether fibronectin-collagen binding interactions are required for cell proliferation or tissue self-assembly, studies were conducted using the inhibitory peptide, R1R2, a peptide derived from the bacterial adhesin SFS, that has sequence homology to collagen [29]. R1R2 binds to fibronectin [29] and inhibits the binding of fibronectin to native and denatured collagens I, II, and III [26]. Previous work demonstrated that R1R2 inhibits fibronectin-mediated assembly of ECM collagen I fibrils, but does not affect collagen III assembly [26]. We first tested the effects of R1R2 on fibronectin-induced cell proliferation. FN-MEFs seeded on polymerized collagen gels in the absence of fibronectin do not proliferate [21] and remain rounded over the course of 6 days (Fig. 6Ba,f). The increase in cell proliferation that occurs in response to either 25 nM or 100 nM fibronectin was completely inhibited by addition of R1R2 (Figure 6A), and was not affected by addition of the control peptide, III-11C (Figure 6A), demonstrating specificity of the R1R2 fragment. Neither R1R2 nor III-11C reduced cell numbers in the absence of fibronectin (Figure 6A). These data indicate that binding of fibronectin to collagen is required for ECM fibronectin-induced proliferation of cells that are adherent to native collagen substrates.

The effect of blocking fibronectin-collagen binding on cellular self-assembly was assessed next. Interestingly, addition of R1R2 to fibronectin-treated samples did not block cellular self-assembly but instead, shifted day 2 morphologies from discontinuous cell sheets (Fig. 6Bb, d) to 3D spheroids (Fig. 6Bc, e). Differences in microtissue morphology were also observed on day 6, where addition of R1R2 to 25 nM or 100 nM fibronectin-treated cells also resulted in the formation of spheroids (Fig. 6Bh, j) rather than 3D cell aggregates (Fig. 6Bg) or dense cell sheets (Fig 6Bi). Thus, binding of fibronectin to collagen contributes to the morphology of multicellular structures, but is not required for fibronectin-induced intercellular cohesion.

### Fibronectin-Collagen Co-assembly and Microtissue Morphology

**Figure 7:**
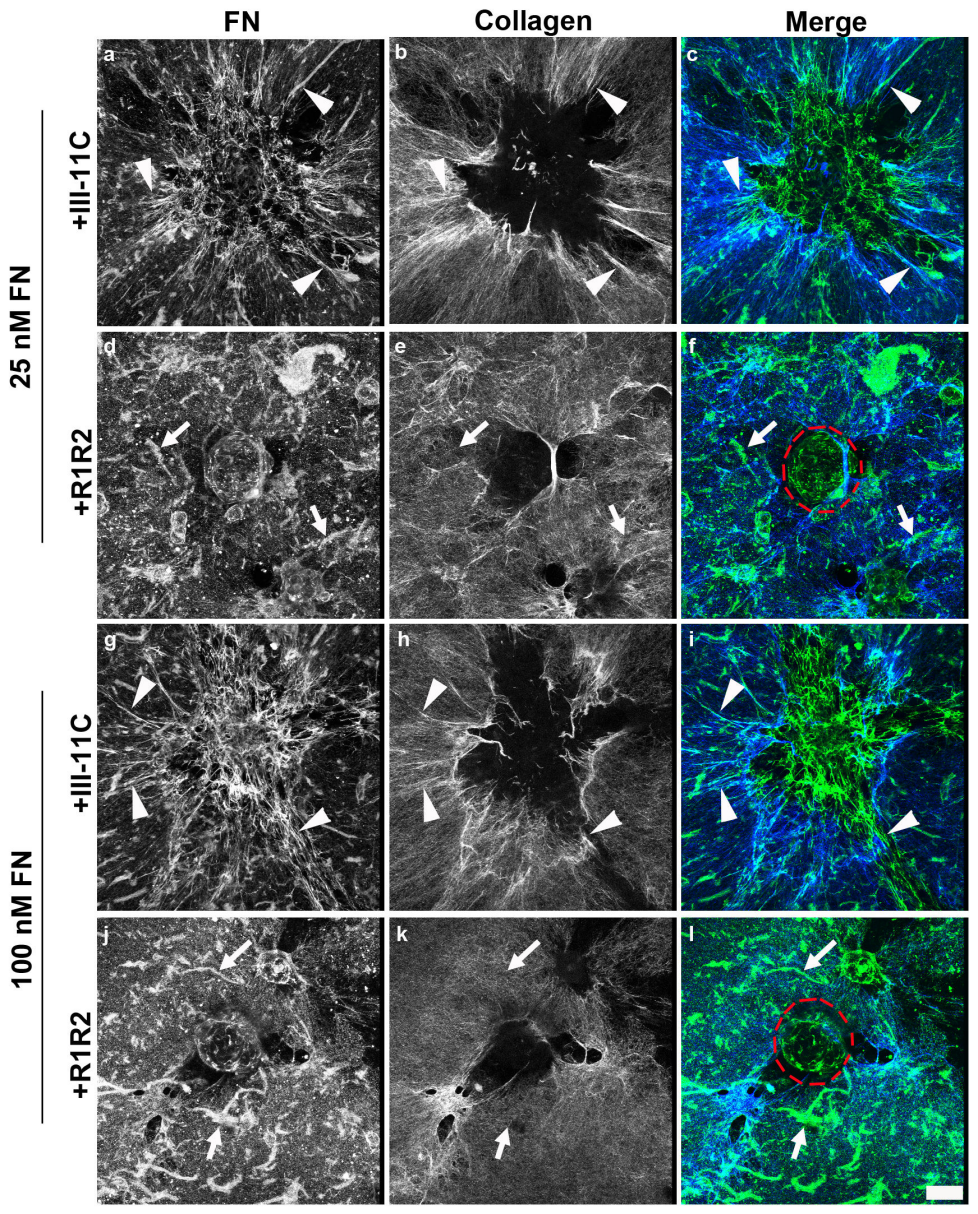
Collagen-fibronectin binding is required for ECM fibril organization. FN-null MEFs adherent to polymerized collagen were treated with fibronectin (25 or 100 nM) in the presence of either R1R2 or the control peptide III-11C. Following a 4-day incubation, cells were processed for immunofluorescence microscopy. Fibronectin (green) and collagen (blue) were visualized using an anti-FN pAb and second harmonic generation imaging, respectively. Images were collected at and above the collagen substrate and then projected onto the z-plane using ImageJ. Dashed red lines denote spheroid location. Arrowheads indicate colocalizing fibronectin and collagen fibrils. Arrows show fibronectin fibrils that do not colocalize with collagen fibrils. Scale bar, 50 μm.

**Figure 8:**
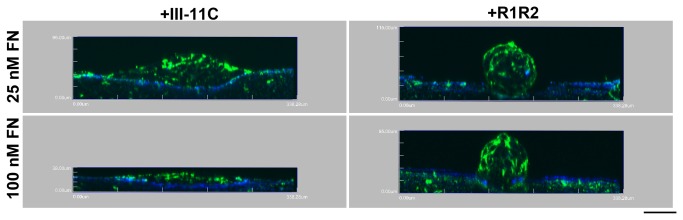
Microtissue morphology depends on fibronectin-collagen binding. Collected images of 4-day microtissues formed in response to either 25 nM or 100 nM fibronectin (FN), pretreated with either R1R2 or III-11C, were reconstructed in 3D and projected onto the x-y plane. Fibronectin (green) and collagen (blue) are shown. Scale bar, 50 μm.

To examine further the role of collagen-fibronectin co-assembly in microtissue morphology, we assessed whether fibronectin-collagen binding interactions play a role in determining the structural pattern of ECM fibrils. Collagen-adherent FN-null cells were treated with fibronectin in the presence of either R1R2 or III-11C and cultured for 4 days. Fibronectin and collagen fibrils were visualized simultaneously using two-photon and second harmonic generation imaging, respectively. Similar to the day 2 images shown in Figure 4, elongated fibronectin fibrils (Figure 7a; arrowheads) that co-localized with collagen fibers (Figure 7b, c; arrowheads) were assembled at the periphery of 3D aggregates formed in response to 25 nM fibronectin pretreated with the control peptide, III-11C. In contrast, addition of R1R2 to block binding of fibronectin to collagen resulted in disorganized fibronectin fibrils (Figure 7d; arrows) and reduced cell-mediated collagen remodeling (Figure 7e; arrows). Similar inhibitory effects of R1R2 on fibronectin fibril organization (Figure 7j) and collagen remodeling (Figure 7k) were observed in the presence of 100 nM fibronectin. *En face* (Figure 7c, f, i, l) and side projection images (Figure 8) demonstrate that addition of R1R2 to fibronectin-treated cultures similarly affected microtissue morphology at either fibronectin concentrations, resulting in the formation of symmetrical 3D spheroids (Figures 7f, l and 8) that contained pericellular fibronectin fibrils (Figure 8).

**Figure 9:**
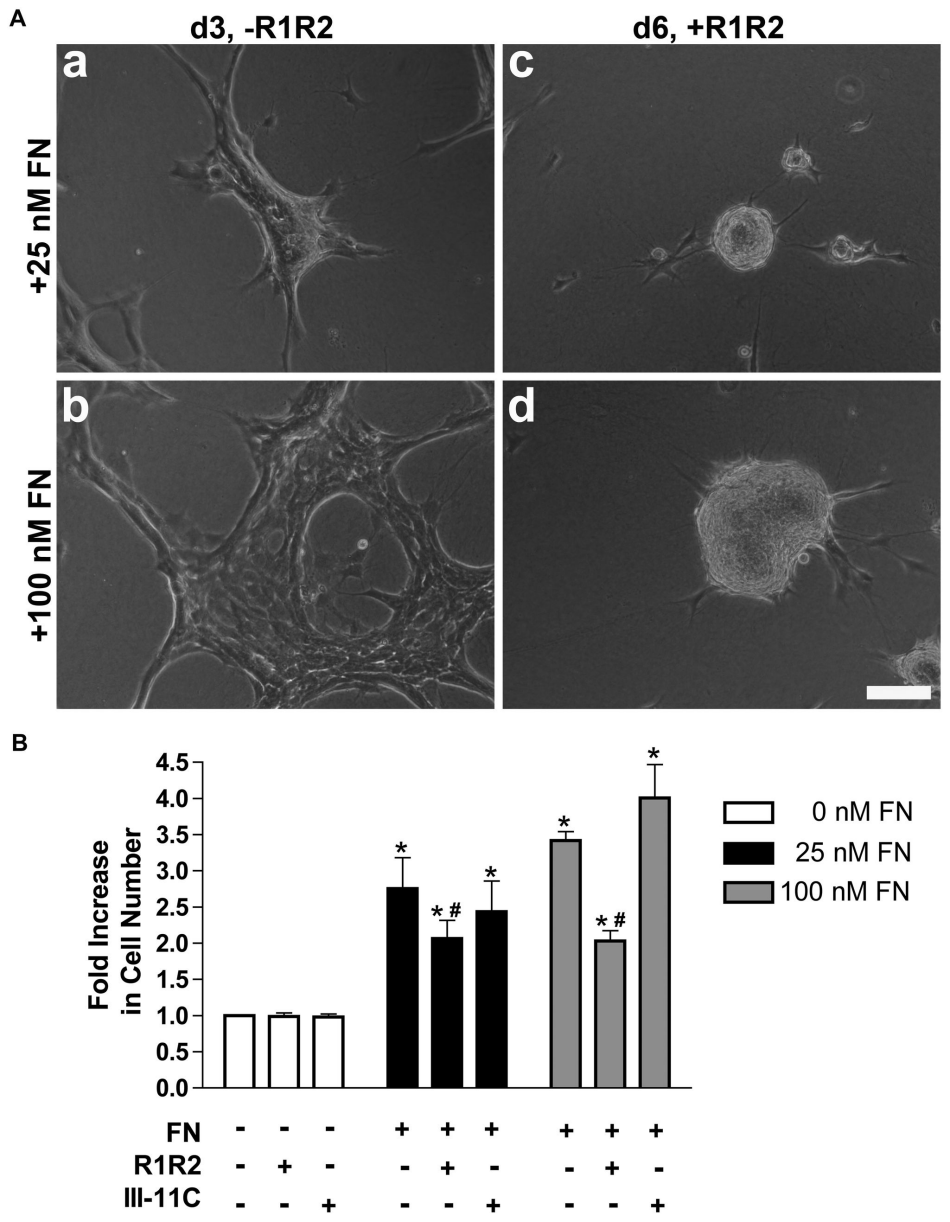
Disrupting collagen-fibronectin binding alters microtissue shape and reduces cell proliferation. Collagen-adherent FN-null MEFs were treated with 25 or 100 nM fibronectin. Following a 3-day incubation, media were removed and replaced with media containing fibronectin (25 or 100 nM) pretreated with R1R2 or III-11C. Cells were then incubated an additional 3 days. (A) Phase contrast images were collected on day 3 prior to treatment with R1R2 (a,b) and again on day 6 (c,d). Images represent 1 of 3 experiments performed in triplicate. Scale bar, 100 μm. (B) Cell number was determined on day 6 using MTT. Data are presented as fold increase in cell number versus non-treated (0 nM FN) controls + SEM of 3 experiments performed in triplicate. *Significant vs. ‘-FN’, *p*<0.001 (ANOVA). ^#^Significant vs. respective ‘+FN’ and ‘+FN/III-11C’, *p*<0.001 (ANOVA).

To determine whether continuous fibronectin-collagen binding interactions are required to maintain microtissue shape or fibronectin-stimulated cell proliferation, microtissues were first allowed to form for 3 days (Fig. 9Aa, b). Media were then removed and replaced with fresh media containing fibronectin and either R1R2 or III-11C peptides. In both cases, addition of R1R2 to either 3D aggregates (Fig. 9Aa) or cell sheets (Fig. 9Ab) triggered a morphological transition to 3D spheroids (Fig. 9Ac, d). Interestingly, addition of R1R2 to preformed 3D aggregates produced numerous small-diameter spheroids (Fig. 9Ac), while addition of R1R2 to preformed cell sheets produced spheroids with larger diameters (Fig. 9Ad). Moreover, the increases in cell number induced by fibronectin over the full course of 6 days were inhibited by addition of R1R2 on day 3 (Figure 9B). Together, these data indicate that binding of fibronectin to collagen is required to maintain microtissue morphologies and fibronectin-mediated cell proliferation. Disruption of collagen-fibronectin interactions after microtissues have formed triggers changes in microtissue shape and disrupts fibronectin-induced cell proliferation.

## Discussion

We demonstrated previously that fibronectin matrix polymerization stimulates the process of self-assembly when cells are adherent to native collagen substrates [21]. The extent of fibronectin-induced cell proliferation and the ultimate shape of microtissues formed in response to fibronectin were influenced by both fibronectin and collagen concentrations [21]. We now report that fibronectin binding to collagen is necessary for collagen fibril remodeling and allows for fibronectin fibril extension. Collagen-fibronectin fibril co-assembly is necessary for fibronectin-induced cell proliferation and determines 3D microtissue morphology. Moreover, continual fibronectin-collagen interactions are required to maintain 3D microtissue shape and ECM fibronectin-mediated cell proliferation. In contrast, the binding of collagen to fibronectin is not required for fibronectin-induced intercellular cohesion.

Previous studies showed that fibronectin matrix assembly can promote intercellular cohesion of cells in hanging drop cultures [19]. In those studies, fibronectin localized to regions of cell-cell contacts in a manner similar to that observed in the current study, where pericellular fibronectin was observed within the central region of 3D cellular aggregates (Figure 2B). Others have shown that decreasing substrate adhesivity increases cell-cell adhesions [36]. In the present study, blocking fibronectin-collagen interactions promoted cell cohesion into 3D spheroids that contained pericellular fibronectin (Figure 8). Together, these data suggest that blocking the formation of fibrillar fibronectin-collagen extensions onto the substrate favors fibronectin-enriched cell-cell contact formation. Fibronectin matrix assembly is required for cellular self-assembly [21] and only pericellular fibronectin was observed in the 3D spheroids (Figure 8), providing evidence that pericellular fibronectin is an ECM-form of fibronectin despite the absence of extensive actin stress fibers (Figure 2A; d4, 25 nM FN). ECM fibronectin can stimulate cell proliferation [16]. However, in the present study, pericellular ECM fibronectin was not associated with cell proliferation (Figure 3A, B), suggesting that pericellular fibronectin is a functionally distinct pool of ECM fibronectin that supports cell cohesion and tissue self-assembly, but not cell proliferation.

Fibronectin and actin underwent similar patterns of spatial and temporal reorganization within microtissues during the transition from 2D cell sheets to 3D aggregates. Within 3D aggregates, proliferating cells and actin stress fibers were organized spatially as a function of soluble fibronectin concentration. In particular, the transition from 2D to 3D was associated with the development of regional differences in cell proliferation, with proliferating cells confined to the periphery of the multicellular structures. Regional differences in fibronectin and collagen co-assembly were also observed within self-organizing cellular networks (Figure 4) and were observed on day 2, prior to the spatial shift in proliferating cells.

The mechanism by which fibronectin-collagen co-assembly stimulates cell proliferation is currently unknown. ECM fibronectin-specific effects on cell behaviors, including proliferation, migration, and contractility, are mediated, in part, through the exposure of a matricryptic heparin-binding domain that is normally buried within the first type III repeat of fibronectin (FNIII-1) [17,37,38]. Thus, as one possibility, the interaction of fibronectin with collagen during co-assembly may directly induce a conformational change in FNIII-1 to expose the matricryptic growth-promoting site. In the present study, proliferating cells were observed throughout the cell sheets on day 2 (Fig. 3Aa, b). In contrast, side-projection images of day 2 cell sheets show central regions where there was a clear spatial separation between collagen staining on the basal surface and fibronectin staining on the apical surface (Figure 5). These data suggest that fibronectin-mediated cell proliferation is not due to the direct interaction of cells with co-assembled collagen-fibronectin fibrils. Rather, fibronectin-collagen fibrils at the periphery of these multicellular structures (Figure 5, arrows) may serve as anchoring filaments that resist cell tension generated in response to fibronectin matrix assembly [18] to allow for fibronectin fibril extension [39] and in turn, exposure of the matricryptic site at regions distal to the collagen-fibronectin tethers.

The development of tension in the organizing cell sheets can be seen indirectly by the presence of actin stress fibers (Figure 1B) and radially aligned fibronectin and collagen fibrils that connect to the substrate surface (Figures 4 and 5). Cell-derived tension on anchored nascent fibronectin fibrils would allow for the formation of extended fibronectin fibrils [39], as observed at the periphery of the 3D aggregates (Figure 2B, b) and throughout the day 2 and day 4 cell sheets (Figure 2A). The elongated fibronectin fibrils and associated actin stress fibers that formed indirectly as a consequence of these collagen-fibronectin tethers were associated with active cell proliferation (Figure 2A). Disrupting fibronectin-collagen fibrils within preformed microtissues caused the discontinuous cell sheets to coalesce into 3D spheroids (Figure 9A), indicating that the R1R2 blocking peptide disrupted the collagen-fibronectin anchoring filaments and released the tension on the extended ECM fibrils.

Blocking fibronectin-collagen interactions with the R1R2 peptide also inhibited fibronectin-induced cell proliferation (Figures 6 and 9). Previous work demonstrated that fibronectin-collagen interactions are essential for cell migration on collagen-coated substrates, and for fibronectin-induced collagen gel contraction [26]. Exposure of matricryptic sites in fibronectin is thought to depend on the application of tensional forces on fibronectin molecules [40,41,42]. Thus, cell-mediated tensional forces applied to fibronectin fibrils anchored to the substrate via fibronectin-collagen bundles may extend fibronectin fibrils to expose the growth-promoting site within FNIII-1 to stimulate cell proliferation. In this way, cell proliferation is confined to regions having extended fibronectin fibrils, as observed in Figure 2, allowing for regional variations in fibronectin fibril function.

The FN-null cell model has proven to be a valuable tool for determining the behavior of cells in the complete absence of fibronectin and for identifying a role for the fibrillar ECM form of fibronectin in a number of cell behaviors, including proliferation [16], spreading [37], migration [17], contractility [18], cellular self-assembly [21] ECM organization [23,43], and matrix turn-over [44]. Importantly, the effects of ECM fibronectin have not been limited to FN-null cells, as we and others have subsequently observed similar ECM fibronectin-dependent responses in a variety of other cell types, including small airway epithelial cells [17], dermal fibroblasts [18,35], aortic smooth muscle cells [23,45], mesenchymal stem cells [28] and in tissues in situ [15,46]. As such, results obtained using the FN-null cell model have been strongly predictive of fibronectin-dependent behavior in fibronectin-expressing cells.

**Figure 10:**
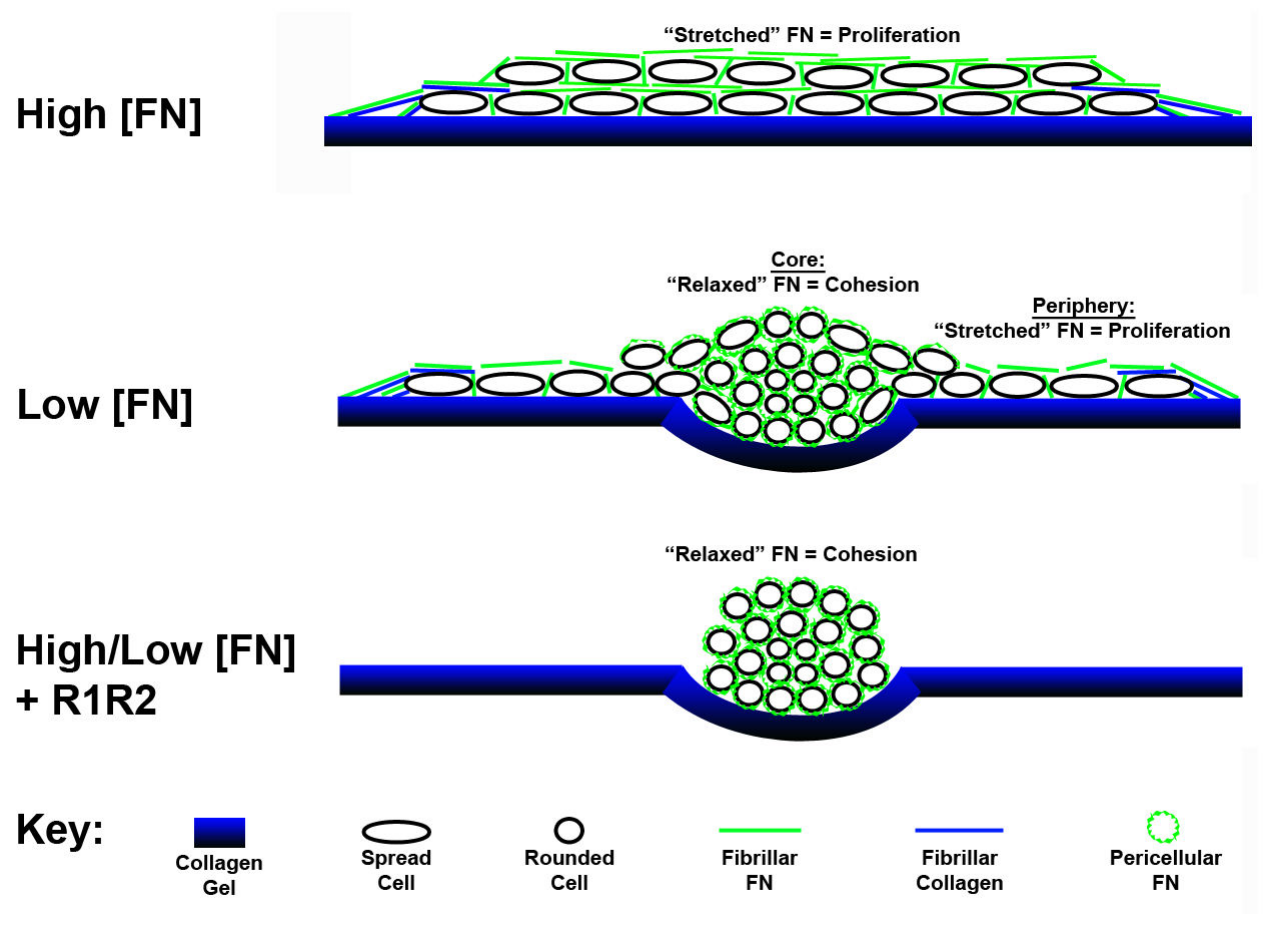
Proposed role of fibronectin-collagen fibrils in tissue self-assembly. Schematic representation of collagen-fibronectin co-assembly in the development of regional differences in cell proliferation and intercellular cohesion. Fibronectin matrix assembly by cells adherent to native collagen type I substrates stimulates cell network formation and cell contraction. Co-assembled collagen-fibronectin bundles provide anchors for cell networks to develop isometric tension and extended fibronectin fibrils that promote cell proliferation.

A model that describes a role for fibronectin-collagen fibril interactions in tissue self-assembly is shown in Figure 10. In the presence of high concentrations of soluble fibronectin, cells adherent to native collagen gels polymerize a dense network of fibronectin and collagen fibrils. Collagen-fibronectin tethers anchor the cell sheet to the collagen substrate, and allow for isometric tension generation and assembly of extended (“stretched”) fibronectin fibrils throughout the multicellular structure. Exposure of growth-promoting matricryptic sites in extended fibronectin fibrils stimulates cell proliferation [37,38]. Fewer or thinner collagen-fibronectin fibrils formed in the presence of lower concentrations of soluble fibronectin offer less resistance to cell contractile forces, leading to compaction of the central core of cells and relaxation of fibronectin fibrils within this region. The central, relaxed fibronectin fibrils mediate intercellular cohesion, while the peripheral, extended fibrils stimulate cell proliferation. The R1R2 blocking peptide releases isometric tension by disrupting preformed collagen-fibronectin bundles, causing cell sheets to condense into 3D spheroids that remain held together by relaxed fibronectin fibrils that do not signal cell proliferation. Similarly, blocking fibronectin-collagen fibrils from forming prevents anchoring of fibronectin fibrils with the collagen substrate, leading to the formation of relaxed fibronectin fibrils that mediate intercellular cohesion, but not proliferation.

In summary, spatial variations in fibronectin-collagen co-assembly and tethering of fibronectin-collagen fibrils to the underlying substrate can lead to regional differences in fibronectin fibril structure and actin organization, and in turn, the spatial separation of proliferation signals. Further, cell proliferation and microtissue shape can be temporally controlled with a peptide that blocks collagen-fibronectin binding interactions. These data provide evidence that the transition of ECM fibronectin from a structural role to a signaling one depends on the co-assembly of collagen fibrils and the subsequent application of tension on anchored fibronectin fibrils.
